# Transparent and complete reporting of confounding in observational research

**DOI:** 10.1002/jcv2.12086

**Published:** 2022-06-02

**Authors:** Henrik Larsson

**Affiliations:** ^1^ School of Medical Sciences Örebro University Örebro Sweden

**Keywords:** confounding, observational research, reporting guidelines, risk factors

## Abstract

Improved understanding of causal risk factors for child and adolescent mental health problems are dependent on observational research. Although confounding is a major limitation of observational research, this problem is widely ignored in the reporting and dissemination of findings from observational studies in psychiatric journals. There is clearly a need for improved reporting of confounding and more careful interpretation of observational research in psychiatry.

As randomized clinical trials, the gold standard approach for addressing confounding and establishing causal effects, are unfeasible or unethical for most putative risk factors, advances in the causal understanding of risk factors for child and adolescent mental health problems are dependent on observational research (Ohlsson & Kendler, 2020). Recent research have indicated poor quality of reporting of confounding in observational studies published in psychiatric journals (Munkholm et al., 2020; Pouwels et al., 2016). This is problematic given that bias from confounding is a major potential limitation in observational research. The aim of this editorial is to (i) highlight the main findings from two publications in the June issue of JCPP Advances, that both used prospective, observational research designs to explore associations between early risk factors and child and adolescent mental health problems and (ii) to discuss these findings in the context of transparent and complete reporting of confounding.

## REPORTING GUIDELINES

The internationally recognised EQUATOR Network (https://www.equator‐network.org/about‐us/uk‐equator‐centre/) works to improve the reliability and value of publications of research through transparent and complete reporting. The EQUATOR Network have developed reporting guidelines for all of the main study designs in health research, such as the Consolidated Standards of Reporting Trials (CONSORT) statement, the Preferred Reporting Items for Systematic Reviews and Meta‐analyses (PRISMA) statement and the Transparent Reporting of a multivariable prediction models for Individual Prognosis Or Diagnosis (TRIPOD)  statement.

As exemplified in the reporting of a pilot controlled trial of an integrated care pathway for depression by Courtney et al. (2022) in the June issue of JCPP Advances, our journal requires authors of randomized trails to conform to the CONSORT statement. JCPP Advances also requires authors to adhere to the PRISMA guideline in the reporting of systematic reviews. The June issue of JCPP Advances have included three high quality systematic reviews covering important research questions related to (i) structural and functional brain changes associated with peer‐victimisation, bullying, and cyberbullying (Ke et al., 2022), (ii) the associations between adverse experiences and mental health outcomes in individuals with minority identities related to sexual orientation, gender expression, or gender identity (Jonas et al., 2022), and (iii) the use of exposure in the management of anxiety‐related disorders among young people (Teunisse et al., 2022). As expected from publications in JCPP Advances, the above mentioned systematic reviews all followed PRISMA guidelines. Importantly, protocols were pre‐registered, several databases were used to identify relevant studies, at least two reviewers independently screened each record and the results of the search and selection process were described in a PRISMA flow diagram.

Strengthening the Reporting of Observational Studies in Epidemiology (STROBE) is another major reporting guideline that has been developed by the EQUATOR Network for the reporting of observational research. Because bias due to confounding is a major limitation of observational research, the STROBE checklist includes multiple items related to confounding (e.g., “all potential confounders are clearly defined”, “all statistical methods, including those used to control for confounding are described” and “reporting of unadjusted estimates and, if applicable, confounder‐adjusted estimates and their precision together with a clear description of the confounders that were adjusted for and why they were included”).

## TRANSPARENT AND COMPLETE REPORTING OF CONFOUNDING IN OBSERVATIONAL STUDIES

Recent research have examined the reporting of confounding in observational studies (Munkholm et al., 2020; Pouwels et al., 2016). One of these studies selected a random sample of 120 articles from five psychiatric specialty journals and evaluated how confounding was considered in the reporting of the discussion and abstract (Munkholm et al., 2020). The study found that the term ‘‘confounding’’ was mentioned in the abstract or discussion in 55% of the included papers, while the term ‘‘bias’’ was highlighted in 57% of the included articles. Only about 20% of the papers acknowledged residual confounding and very few expressed any caution in relation to confounding or other bias in their conclusions or in the abstract. These findings are overall in agreement with previous reports of poor quality of reporting of confounding in observational studies published in general medical, medical specialty, and epidemiology journals (Pouwels et al., 2016).

## OBSERVATIONAL STUDIES IN THE JUNE ISSUE OF JCPP ADVANCES

Two papers in the June issue of JCPP Advances represent useful examples of how observational studies can explore theory‐driven, biologically plausible hypothesis about risk factors and help identify the mechanisms through which risk factors are associated with child and adolescent mental health problems. The publication by Walle et al. (2022), in the June issue of JCPP Advances, explored maternal infections during pregnancy as a risk factor for Attention‐deficit hyperactivity disorder (ADHD) in offspring. They used data from the prospective Norwegian Mother, Father and Child Cohort Study (MoBa), including more than 112,000 pregnancies, linked with data from the Medical Birth Registry of Norway and the Norwegian Patient Registry. The large sample size allowed the authors to investigate the potential role of specific groups of prenatal maternal infections, timing of exposure and the role of fever. The main findings suggest that prenatal exposure to maternal infections, particularly with co‐occurring episodes of fever, are risk factors for ADHD and that type of infection and timing of exposure might influence the associations.

The study by Fish‐Williamson et al. (2022) provides insight into the relationship between prenatal antibiotic exposure in pregnancy and childhood socioemotional developmental outcomes. This article used data from the prospective Growing Up in New Zealand Study (GUiNZ), which prospectively follows children starting in the last trimester of pregnancy into early childhood. A national comprehensive pharmaceutical database was used to determine children's prenatal antibiotic exposure, while socioemotional development was measured using a composite score derived from several commonly used socioemotional tasks administered between 9 months and 4.5 years of child age. This study found that socioemotional development was not associated with prenatal antibiotic exposure at any dosage or trimester of pregnancy after statistically adjusting for important confounding factors.

In contrast to many publications in psychiatric specialty journals (Munkholm et al., 2020), the two observational studies discussed above put considerable efforts into transparent and complete reporting of confounding. Even though none of the two studies stated use of a reporting guideline, most STROBE items related to confounding were actually carefully considered. Consistent with STROBE, both studies provided a clear rational and motivation for the selection of statistical covariates and all confounders and statistical models were clearly defined. The above mentioned observational studies also presented both unadjusted and confounder‐adjusted estimates along with their precision, which is recommended by STROBE. The study by Walle et al. (2022) expressed caution in relation to confounding in the abstract and also highlighted that the small effect sizes require careful interpretations. Importantly, the study emphasized that associations between maternal infections during pregnancy and ADHD in offspring needs to be explored using other study designs, such as negative controls, cross‐contextual designs, instrumental variables (e.g., Mendelian randomization), family‐based studies and natural experiments. The use of such methods enables researchers to rigorously test competing, theory‐driven hypotheses and help identify the mechanisms through which risk factors are associated with child and adolescent mental health problems.

## CONCLUSIONS AND FUTURE DIRECTIONS

Reports of research should provide clarity around which questions were addressed and why, what was done, what was shown, and what the findings mean. Even though reporting quality of a study does not necessarily reflect the methodological quality of the study, transparent and complete reporting is needed to allow readers to evaluate the strengths and limitations of the study and to replicate the study and also to conduct evidence‐synthesis studies. Insufficient reporting, therefore, hampers possibilities to effectively translate research into improved care for young people with mental health problems.

Most readers of JCPP Advances probably agree that the identification and discussion of the limitations of a study are a critical aspect of reporting. Although confounding is a major limitation of observational research it is widely ignored in the reporting and dissemination of findings from observational studies in psychiatric journals (Munkholm et al., 2020). There is clearly a need for improved reporting of confounding and more careful interpretation of observational research in psychiatric journals.

## AUTHOR CONTRIBUTION

**Henrik Larsson:** Conceptualization; Writing – original draft; Writing – review & editing.

## CONFLICTS OF INTEREST

Henrik Larsson reports receiving grants from Shire Pharmaceuticals; personal fees from and serving as a speaker for Medice, Shire/Takeda Pharmaceuticals and Evolan Pharma AB outside the submitted work; and sponsorship for a conference on attention‐deficit/hyperactivity disorder from Shire Pharmaceuticals outside the submitted work. He is the Editor‐in‐Chief for JCPP *Advances*.
